# Akinete formation under nitrogen limitation in an invasive cyanobacterium

**DOI:** 10.3389/fmicb.2025.1677844

**Published:** 2025-12-12

**Authors:** Catalina Ríos-Henríquez, Guntram Weithoff

**Affiliations:** Aquatic Ecology Group, Institute of Biochemistry and Biology, University of Potsdam, Potsdam, Germany

**Keywords:** Nostocales, *Raphidiopsis raciborskii*, *Cylindrospermopsis*, resting stage, strain-specificity, benthic

## Abstract

Invasive species are a global problem threatening the function of ecosystems. Besides prominent species, microbial invaders are considered as “invisible” and have spread over almost all continents. For such invisible, invasive cyanobacteria, akinete formation plays a key role in species dispersal, establishment and persistence in new ecosystems. *Raphidiopsis raciborskii* (Nostocales) is a successful invader of temperate ecosystems originating from the tropics that forms akinetes in their new habitats to overcome unfavorable winter conditions. In this study, we investigated akinete formation in *R. raciborskii* as a response to nitrogen limitation by exposing four genetically distinct strains to nitrogen-free medium. Cultures were maintained for 30 days at two temperature regimes, 20 °C (representing typical culture conditions) and 28 °C (mimicking hot summer conditions). All four strains started akinete formation within 3 days. However, we observed significant intraspecific variation in response to temperature, including differences in akinete abundance, maturation and size. Notably, vegetative cells continued to grow while akinetes were being formed, indicating the ability of *R. raciborskii* to simultaneously maintain growth and investment into dormancy. The pronounced strain-specific variation may provide adaptive advantages, enhancing the capacity of *R. raciborskii* to colonize diverse environments. Our results extend the concept of high intraspecific variability from the planktonic to the benthic phase. Understanding strain-specific dormancy strategies is crucial for predicting the ecological success and persistence of cyanobacteria under changing environmental conditions. These findings have important implications for bloom frequency, invasion dynamics, and the long-term establishment of these often-overlooked microbial invaders.

## 1 Introduction

Biological invasions are considered among the greatest threats to ecosystems, by promoting diversity loss, altering community composition, and generating economic costs (Pyšek et al., 2020; Sala et al., 2000). We refer to *invaders* in an ecological sense – that is, species that expand and establish beyond their historical or previously documented range, regardless of whether their spread is human – mediated or naturally driven. This definition emphasizes contemporary range expansion and ecological impacts rather than on deep-time evolutionary dispersal. In Europe, non-native species such as the zebra mussel (*Dreissena polymorpha*, Strayer, 2009) or signal crayfish (*Pacifastacus leniusculus*, Holdich et al., 2009) have substantially affected native communities and ecosystem processes. Recent studies in Germany has highlighted additional cases across diverse ecosystems, providing a comprehensive overview of established non-native species and their ecological consequence (Haubrock et al., 2025). Free-living microorganisms are considered as invisible invaders, because their invasion process is subtle, less evident, and more difficult to detect than in macroscopic organisms (Litchman, 2010). Cyanobacteria are one group of such invisible invaders, as their establishment and proliferation often occur through cryptic stages. In particular, their persistent dormant cells (akinetes) form an overlooked propagule bank that enables colonization and seasonal re-establishment without being visually detectable. By focusing on the dynamics of akinetes, our study addresses this cryptic phase of cyanobacterial invasion and provides mechanistic insight into how these “invisible invaders” initiate and sustain their presence in aquatic ecosystems.

Over recent decades, Cyanobacteria have received increasing attention due to their rapid proliferation in aquatic ecosystems. Driven by climate change and anthropogenic factors, such as temperature increase, alterations in the mixing regime and nutrients enrichment, the dominance of cyanobacteria in aquatic ecosystems is increasing (Cottingham et al., 2021; Woolway et al., 2021). Nevertheless, blooms have also been reported in cold, oligotrophic lakes, although are generally less frequent (Fuentes et al., 2022; Reinl et al., 2023). These occurrences may reflect climate-driven shifts in mixing dynamics, thermal stratification, or ice-cover duration. Bloom patterns also vary among ecosystem, because not all systems exhibit frequent or intensified blooms, and some long-term studies report no evidence of widespread bloom intensification (Wilkinson et al., 2022). This variability likely reflects differences in nutrient regimes, lake types, climate forcing, and monitoring intensity across regions.

Altered abiotic condition scan facilitate the invasion of cyanobacterial into new habitats (Wejnerowski et al., 2020) and subsequent proliferation can have significant effects on the biotic and abiotic characteristics of water bodies (Sukenik et al., 2015). *Raphidiopsis raciborskii* (Aguilera et al., 2018), formerly *Cylindrospermopsis raciborskii* (Seenayya and Subba Raju, 1972; Padisák, 1997), is one of the cyanobacterial species that benefits from climate warming by invading new habitats and expanding its geographic distribution on almost every continent, from tropical towards temperate regions (Alvarez Dalinger et al., 2024; Hamilton et al., 2005; Padisák, 1997; Sinha et al., 2012; Wood et al., 2014). Most studies on cyanobacterial invasions have focused on the planktonic phase and several specific environmental factors have been proposed to facilitate invasions for example, a low N: P ratio and high temperature (Smith, 1983; Sukenik et al., 2015). The physiological plasticity of *R. raciborskii*, and its broad temperature tolerance ranging from 11 to 35 °C (Chonudomkul et al., 2004; Dokulil, 2016), allows populations to persist under suboptimal conditions and colonize temperate and occasionally cooler environments. However, the role of akinetes, the resting cells of Nostocalean cyanobacteria, have been widely understudied. Once the invasion process has begun, akinetes ensure the survival during unfavorable conditions such as winter in temperate regions, thereby contributing to the species´ ability to persist and adapt (Chonudomkul et al., 2004; Cottingham et al., 2021; Ramm et al., 2017; Sukenik et al., 2012). This feature plays a key role in the seasonal life cycle of Nostocales combining the planktonic and the benthic phase (Singh et al., 2020): In the planktonic phase, vegetative growth in temperate water bodies is often highest at high summer temperatures. When light, temperature and nutrient availability decrease at the end of the summer, conditions become unfavorable and the formation of akinetes starts (Cottingham et al., 2021; Kaplan-Levy et al., 2010). During this process, akinetes accumulate a greater amount of glycogen and 8-fold higher granules of cyanophycin compared to vegetative cells (Adams and Duggan, 1999; Singh et al., 2020). The process ends when the akinete has developed a multilayered extracellular envelope, compounded by exopolysaccharides and glycolipids (Perez et al., 2016). Finally, the vegetative population declines, the mature akinetes detach from the filament (Mehnert et al., 2014) and sink to the bottom. Thus, the benthic phase serves as a “seed bank” and akinetes can remain viable for hundreds and even thousands of years at dark and cold conditions (Legrand et al., 2019). After germination, the planktonic phase starts with vegetative cell growth (Kaplan-Levy et al., 2010). In addition, akinete can also serve as dispersal units via wind or animal vectors (Padisák et al., 2016).

Since akinetes are an insurance to overcome unfavorable conditions, their formation is most beneficial before conditions deteriorate. On the one hand, nutrients are required to build up akinetes, on the other hand high nutrient levels favor purely vegetative cell growth. Thus, declining nutrients are a signal for deteriorating environmental conditions. Similarly, at low light the energy demand might exceed the demand for growth and akinete formation and at high light conditions, vegetative growth is favored explaining that moderate light levels are best for akinete formation (Ho et al., 2024; Mehnert et al., 2014; Moore et al., 2005). Another triggering factor is temperature. The optimal temperature for akinete formation ranges between 20 and 30 °C (Mehnert et al., 2014; Yamamoto and Shiah, 2014).

One specific characteristics of the invasive *R. raciborskii* is its high intraspecific trait variation (Bolius et al., 2017) showing a wide tolerance range to key environmental factors facilitating their invasion (Bonilla et al., 2012). Recent studies have shown that the invasion success of *R. raciborskii* is highly strain-specific (Bolius et al., 2020; Weithoff and Stefan, 2024) underlining the importance to study several strains (Willis, 2025). However, all these studies focused on the vegetative, planktonic phase.

In this study, we investigate the combination of strain specificity with akinete formation. In detail, we quantified the formation of akinetes after the onset of nitrogen limitation over a period of 30 days at two temperatures simulating eutrophic freshwater lakes. We hypothesize that nitrogen limitation initiates the formation of akinetes differently at different temperatures and that the formation is strain-specific.

## 2 Methods

Since nitrogen becomes limited in late summer in many shallow lakes in invaded temperate regions (Dolman et al., 2016), we used nitrogen (N) starvation to induce the formation of akinetes. Based on results from a pilot experiment with different temperatures, we quantified the akinete formation at 20 °C (same temperature as culture conditions) and at 28 °C (about maximal temperature in shallow lakes in hot summers). Under standard, nutrient rich conditions, akinetes occur only very rarely. We studied four genetically different strains from invaded temperate region in NE Germany (26D9, 27F11, MEL07, and ZIE11; Bolius et al., 2017) that were found to have a different invasive potential (Bolius et al., 2020). Prior to the start of the experiment, the four strains were cultured for seven days at 20 °C in Woods Hole medium modified from Guillard and Lorenzen (1972) by omitting organic carbon (except for traces of vitamins) to minimize heterotrophic bacterial growth. Under non-limiting conditions, the medium contained 14 mg N L^−1^ - 1.55 mg P L^−1^, at pH 8. Cultures were maintained in climate-controlled incubators (Minitron, Incubator/Shaker, Infors HT, Switzerland) under a 12 h light: 12 h dark cycle, and a light intensity of 93 ± 3 μmol photons m^−2^ s^−1^.

The experiment started by inoculating each strain in triplicate with an optical density 0.1 (5 cm cuvette, 880 nm, UV Mini 1,240 UV–VIS spectrophotometer, Shimadzu, Kyoto, Japan) in nitrogen-free medium with a high phosphorus supply, in a total volume of 250 mL. Because the filament morphology of *R. raciborskii* prevents reliable conversion to individual cell numbers, we report inoculation biomass as particulate carbon. At the start of the experiment, particulate carbon concentration ranged from 9 to 48 mg C L^−1^ depending on the strain (MEL07: 48 mg C L^−1^; 26D9: 28 mg C L^−1^; 27F11: 37 mg C L^−1^; ZIE11: 9 mg C L^−1^). Cultures were grown in 300 mL glass flasks without shaking or aeration. Then, the 20 °C treatments were kept at their temperature and the other half of the flasks were transferred to 28 °C. The experiment ran for 30 days close to batch culture conditions, with a low dilution rate (~6% every 3 days), exchanging 15 mL of culture with fresh medium to maintain constant volume.

**Figure 1:**
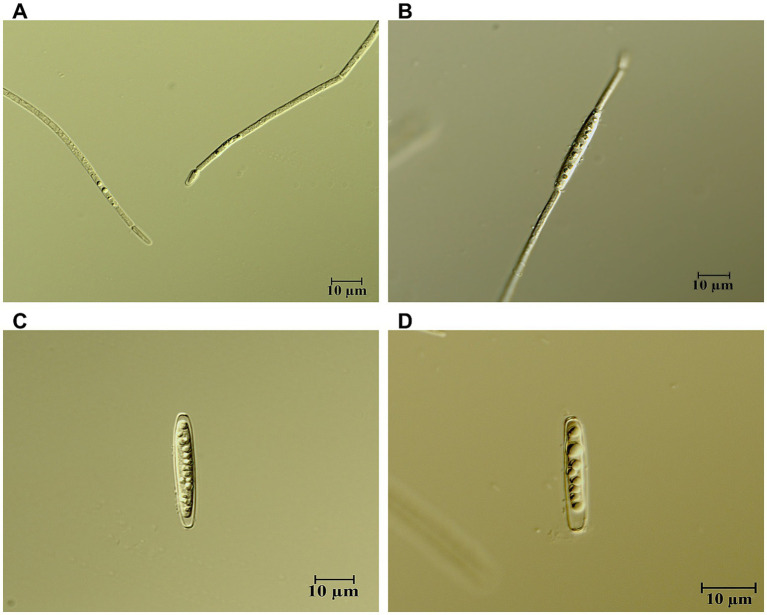
Developmental stages in *Raphidiopsis raciborskii* akinetes. **(A)** Initiation of an akinete. **(B)** Immature akinete within a filament. **(C,D)** Solitary, mature akinete.

We determined particulate carbon and nitrogen concentration on each sampling day after filtering 5 mL of culture onto pre-combusted (at 450 °C) glass fiber filters (GF/C, 25 mm, Whatman International Ltd., Maidstone, UK). After drying, these filters were analyzed using an elementary analyzer (EA 3000, EuroVector S.p. A., Milan, Italy). Chlorophyll concentration was quantified using a PhytoPAM (Photosynthesis and phytoplankton analyzer, Heinz Walz, Germany). The PhytoPAM was calibrated with a dilution series of stock cultures of *R. raciborskii*. Parallel to chl-a measurements with the PhytoPAM, subsamples were filtered on GF/C glass fiber filters, frozen and chl-a was extracted after thawing with hot ethanol. Chl-a was determined using a fluorometer (Turner FD 700, Turner design, Sunnyvale, CA USA) using a calibration curve (Welschmeyer, 1994). Akinete abundance was quantified using four transects per sample by light microscopy using the Utermöhl technique (400x, Primovert, Carl Zeiss, Jena, Germany), on samples preserved with Lugol’s iodine solution. We differentiated between mature and immature akinete: mature akinete were either single cells (not part of a filament) or only attached to a heterocyst, and immature ones were part of the filament (Figure 1). For morphological characterization and size measurements, we used an upright light microscope Axioskop 2 (Carl Zeiss, Jena, Germany) with a camera (Axiocam 506 color, software Zen 2, Carl Zeiss). Akinete abundance was expressed per liter, per mg C and per μg Chlorophyll *a* to capture both ecological abundance and resource allocation strategies.

In order to compare main effects in strain response, we applied a Multivariate Generalized Linear Model (GLM) for testing differences in mean relative, considering the response variables akinetes L^−1^, akinetes mg C^−1^ and akinetes μg Chla^−1^; strains and temperatures as predictors and C: N ratio as a covariate. We only considered the last 3 days of the experiment, i.e., when the abundance of akinetes reached a plateau. Akinete abundance was log_10_-transformed, and the percentage of mature akinetes was square-root, arcsine-transformed to meet the assumptions of normality. Statistical significance was considered at *p* < 0.05. Statistical analyses were performed with SPSS version 29.

## 3 Results

**Figure 2:**
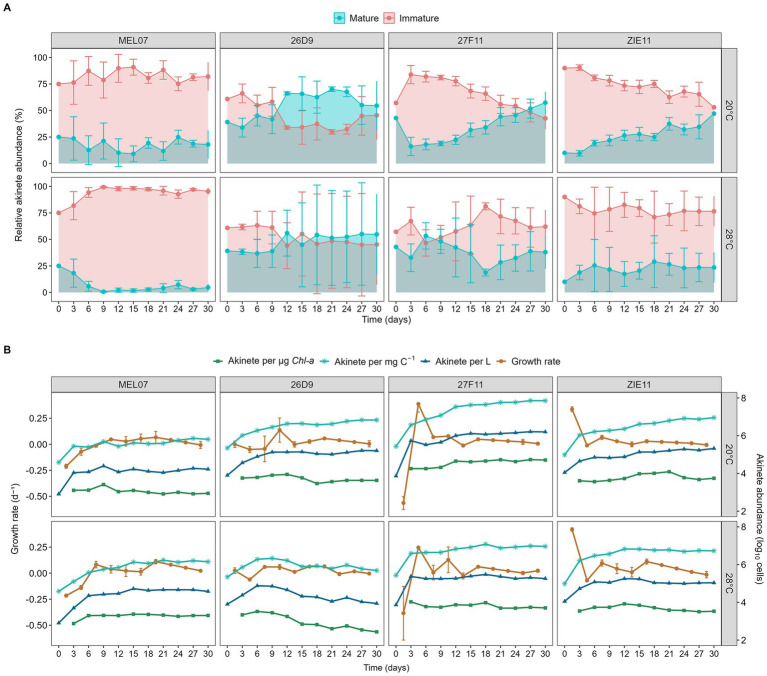
Time course of inter-strain variation in akinete abundance of four strains of *Raphidiopsis raciborskii* (MEL07, 26D9, 27F11, ZIE11) under nitrogen-limited conditions at two temperatures. **(A)** Relative abundance of mature and immature akinetes (%). **(B)** Akinete abundance expressed per liter, per carbon biomass (mg C^−1^) and per chlorophyll (μg Chl-*a*^−1^), together with the corresponding growth rate across treatments to illustrate the coupling between biomass accumulation and differentiation. Values represent mean ± standard deviation. Akinete abundance was log_10_-transformed.

All four strains initiated akinete formation within the first 3 days. We observed differences among strains and temperature in the total abundance of akinetes and the relative share of mature akinetes. The temperature response of the strains also differed underlined by a significant interaction of the two factors (Figure 2 and Table 1).

**Table 1:** Summary of multivariate generalized linear model (GLM) results evaluating the effects of strain and temperature as fixed factors and C: N ratio as co-variant on four response variables: Akinetes L^−1^, Akinetes mg C^−1^, Akinetes μg Chla^−1^, and percentage of mature akinetes (%) in four strains of *Raphidiopsis raciborskii* (26D9, 27F11, MEL07, ZIE11).

Source	Dependent variable	Type III sum of squares	df	Mean square	*F*	Sig.
Corrected model	Akinetes L^−1^	10.133	8	1.267	78.981	**<0.001**
	Akinetes mg C^−1^	10.155	8	1.269	92.625	**<0.001**
	Akinetes μg Chla^−1^	8.942	8	1.118	69.9	**<0.001**
Akinetes mature %	1.126	8	0.141	2.705	**0.046**
Intercept	Akinetes L^−1^	1.014	1	1.014	63.199	**<0.001**
Akinetes mg C^−1^	0.66	1	0.66	48.17	**<0.001**
Akinetes μg Chla^−1^	0.467	1	0.467	29.224	**<0.001**
Akinetes mature %	0	1	0	0.003	0.958
C: N	Akinetes L^−1^	0.012	1	0.012	0.747	0.401
	Akinetes mg C^−1^	0.019	1	0.019	1.396	0.256
	Akinetes μg Chla ^−1^	0.021	1	0.021	1.315	0.269
	Akinetes mature %	0.009	1	0.009	0.179	0.679
	Temperature	Akinetes L^−1^	1.252	1	1.252	78.074	**<0.001**
Akinetes mg C^−1^		1.543	1	1.543	112.577	**<0.001**
Akinetes μg Chl*a* ^−1^		1.465	1	1.465	91.617	**<0.001**
Akinetes mature %		0.149	1	0.149	2.865	0.111
Strain		Akinetes L^−1^	6.753	3	2.251	140.362	**<0.001**
		Akinetes mg C^−1^	6.268	3	2.089	152.456	**<0.001**
		Akinetes μg Chl*a* ^−1^	5.215	3	1.738	108.705	**<0.001**
		Akinetes mature %	0.545	3	0.182	3.488	**0.042**
		Temperature * Strain	Akinetes L^−1^	1.751	3	0.584	36.397	**<0.001**
	Akinetes mg C^−1^		2.05	3	0.683	49.868	**<0.001**
	Akinetes μg Chla^−1^		2.009	3	0.67	41.874	**<0.001**
	Akinetes mature %		0.039	3	0.013	0.251	0.859
	Error		Akinetes L^−1^	0.241	15	0.016		
			Akinetes mg C^−1^	0.206	15	0.014		
			Akinetes μg Chla^−1^	0.24	15	0.016		
			Akinetes mature %	0.781	15	0.052		
			Total	Akinetes L^−1^	1066.688	24			
Akinetes mg C^−1^				606.749	24			
Akinetes μg Chla^−1^				389.809	24			
Akinetes mature %				11.184	24			
Corrected total				Akinetes L^−1^	10.374	23			
				Akinetes mg C^−1^	10.361	23			
				Akinetes μg Chla^−1^	9.182	23			
				Akinetes mature %	1.907	23			
				Akinetes L^−1^: *R*^2^ = 0.929 (Adjusted *R*^2^ = 0.891)						
		Akinetes mg C^-1:^ *R*^2^ = 0.923 (Adjusted *R*^2^ = 0.882)						
		Akinetes μg Chla^−1^: *R*^2^ = 0.891 (Adjusted *R^2^* = 0.833)						
		Akinetes mature %: *R*^2^ = 0.591 (Adjusted *R*^2^ = 0.372)						

The table includes Type III sum of square, degree of freedom (df), Mean square, *F*-value and associated *p*-values. Significant effects (*p* < 0.05) are indicated in bold. Models were fitted using a Gaussian family with log_10_-transformed response variables where appropriate; percentage (%) mature akinetes were square root, arcsine-transformed.

### 3.1 Growth rate

**Figure 3:**
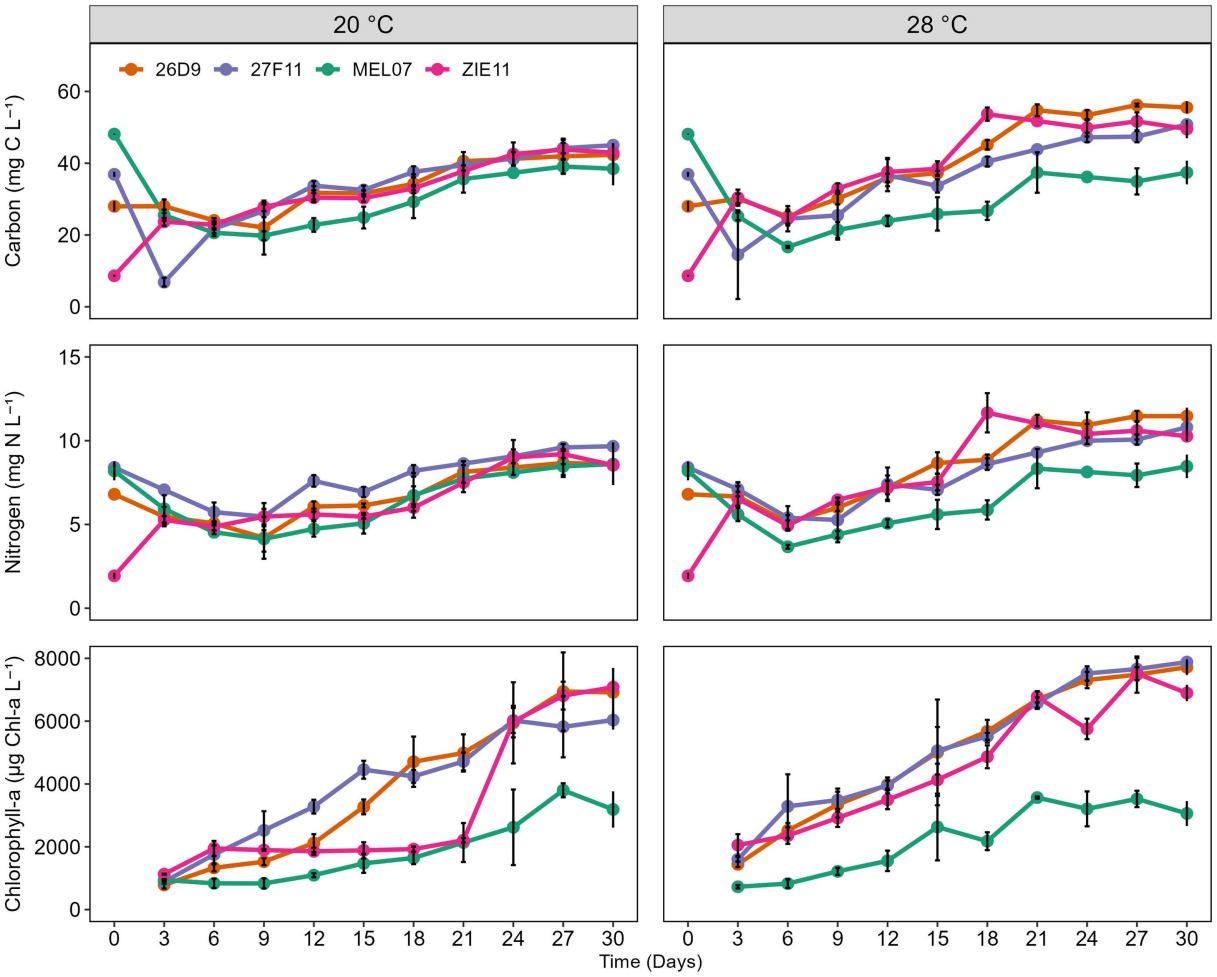
Time course of particulate carbon and nitrogen (mg L^−1^) and chlorophyll *a* (μg L^−1^) in four strains of *Raphidiopsis raciborskii* (MEL07, 26D9, 27F11, ZIE11) under nitrogen-limited conditions at two temperatures. Symbols represent mean values and error bars indicate standard deviations.

After an initial decline in carbon, nitrogen and chlorophyll *a* during the first days of the experiment (Figure 3), all strains grew in the nitrogen-free medium until ca day 18–21, when a plateau was reached. Since the increase in particulate carbon and nitrogen was proportionate, the C: N ratio varied only little throughout the experiment (Figure SM1). Except for strain MEL07, the final concentration of particulate carbon and nitrogen tended to be slightly higher at 28 °C than at 20 °C (Figure 3). The number of akinetes increased not only per liter, but also per unit of carbon demonstrating simultaneous population growth and akinete formation. Akinete abundance rose markedly after the main growth phase and then remained relatively stable, suggesting that akinete formation may be activated once a certain biomass threshold is reached rather than being strictly synchronized with the growth rate (Figure 2B). Chlorophyll concentration increased steeper than carbon resulting in a roughly stable ratio of akinete per chlorophyll (Figures 2, 3; Table SM1).

### 3.2 Akinete formation

After transferring the strains into nitrogen-free medium, all four strains initiated the formation of akinetes exhibiting a higher increase of immature than of mature akinetes. The number of akinetes increased steadily for the following 6 to 18 days reaching a quasi-saturation, except for strain 26D9 at 28 °C. The mean total abundance of akinetes of the last three sampling dates varied among strains by a factor of 93 at 20 °C (MEL07= 8 ± 4.5 · 10^5^ and 27F11 = 7.4 ± 2 · 10^7^ akinetes L^−1^) and by a factor of 18 at 28 °C (26D9 = 5.4 ± 0.9 · 10^5^ and ZIE11 = 9.5 ± 1.5 · 10^6^ akinetes L^−1^) (see Supplementary Table S1). Also, the relative share of mature akinetes was highly variable ranging from 3.5% (MEL07 at 28 °C) to 58% (26D9 at 28 °C). The overall response to the two temperature treatments was strain-specific: Two strains (26D9 and 27F11) produced substantially less akinetes at 28 °C than at 20 °C, in strain MEL07 the opposite pattern was found and in strain ZIE11 no substantial difference was observed (Figure 2).

### 3.3 Morphology

**Figure 4:**
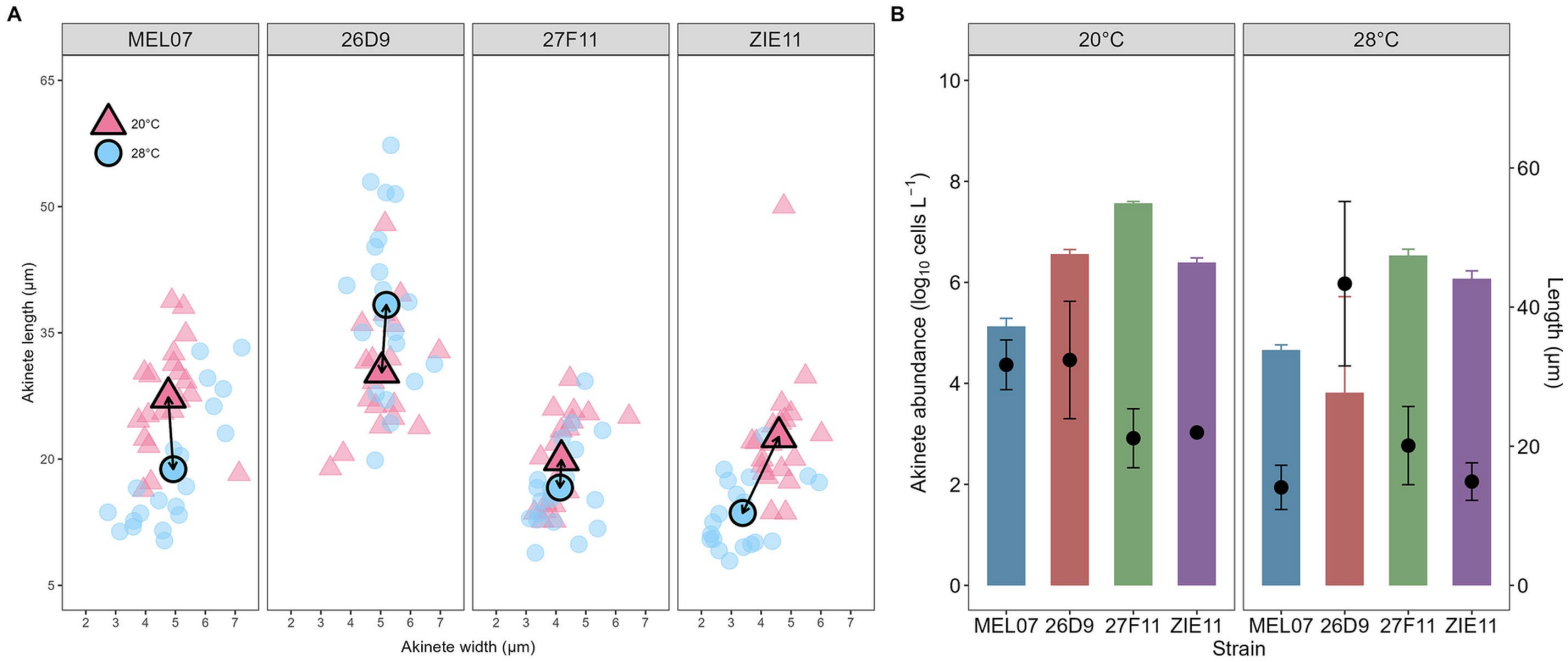
**(A)** Length and width of akinetes at the end of the experiment (nitrogen limitation) at 20 °C (light red triangles) and 28 °C (light blue circles) in four strains of *Raphidiopsis raciborskii* (MEL07, 26D9, 27F11, ZIE11). Bold symbols represent the mean values. **(B)** Mean abundance of mature akinetes (bars) and mean length (black circles) at day 30. Values represent mean ± standard deviation.

The filaments of all four strains are straight shaped, with the heterocyst typically located at the terminal position. In exceptional cases, heterocysts were observed in intercalary positions, and in some filaments, two consecutive heterocyst were present. Immature akinetes were commonly found adjacent to heterocyst, either appearing as solitary or forming short chains of two to five akinetes. In all four strain, mature akinetes retained an oval or elongated shape measuring 19.60–30.73 μm at 20 °C and 17.96–34.74 μm at 28 °C. Akinete length varied among strains and temperature: At 28 °C morphological changes were slightly more pronounced in strain 26D9, MEL07, and ZIE11 compared to those observed at 20 °C. Akinete size of strain 27F11 remained stable across both temperatures. In contrast, strains 26D9 and ZIE11 exhibiting greater morphological alterations at elevated temperatures. Overall, exposure to 28 °C tended to increase the magnitude of morphological change (Figure 4A). We did not find a consistent pattern between akinete abundance and akinete size (Figure 4B).

## 4 Discussion

Significant intraspecific difference in akinete formation (number, size and maturation) was observed among *R. raciborskii* strains. Moreover, this study provides novel evidence of strain-specific simultaneous growth and akinete formation, highlighting the intraspecific variation in survival strategies within species.

### 4.1 Nitrogen limitation as a potential driver for akinete formation

All four *R. raciborskii* strains formed akinetes under nitrogen-limited conditions, initiated formation relatively quickly. Although full parallel nutrient-replete controls were not included within the same experimental series, an independent experiment conducted under comparable conditions with nutrient-rich medium (see Figure SM2) showed low and stable akinete formation. Across all strains, akinete abundance consistently increased under nitrogen limiting conditions. Strain 27F11 exhibited the highest abundance, forming 244 times more akinetes than the control, whereas strains MEL07, 26D9 and ZIE11 showed rather moderate increases, ranging from 2 – to 11 – fold (Figure SM2b). These differences underscore the magnitude of the response, even in the absence of parallel nutrient-replete controls with the same experimental series. These findings support nitrogen limitation as a key inductor for akinete formation in *R. raciborskii*, consistent with previous observations in laboratory and field studies (Mehnert et al., 2014; Sukenik et al., 2015). Nevertheless, the absence of complete nutrient-replete controls across all temperature treatments prevents direct comparison under identical conditions. Future studies including such controls will allow evaluation of the combined effects of nutrient status and temperature on akinete dynamics.

In shallow, temperate lakes, nitrogen is often limited towards the end of the summer while phosphorus remains abundant, resulting in a low N: P ratio (Dolman et al., 2016). Although cyanobacteria require both nutrients for akinete formation, *R. raciborskii* specifically requires a high phosphorus concentration (> 70 μg/L; Rücker et al., 2009). This makes the timing of nitrogen limitation a crucial factor. Under nitrogen limitation, cyanobacteria must differentiate heterocyst for N_2_ fixation — a metabolically expensive process (Turpin et al., 1984; Willis et al., 2016). Since the formation of heterocysts and akinetes involves overlapping metabolic pathways (Zhang et al., 2006), simultaneously producing both structures imposes a high energetic demand. Despite this high demand, our strains were able to form akinetes and fix nitrogen simultaneously shown by a simultaneous increase of akinete abundance and particulate nitrogen (Figures 2, 3). Heterocyst formation may have occurred earlier under nutrient-rich conditions, as Bolius et al. (2017) observed substantial numbers of heterocysts at nitrogen-replete conditions. This suggests an adaptive strategy that supports both nitrogen storage and dormancy. In *Anabaena circinalis*, it was found that during a logistic growth phase, first heterocysts were formed and later akinetes before population growth had stopped (Fay et al., 1984). In our study, *R. raciborskii* strains began to increase akinete formation before day three under nitrogen limitation suggesting a quick perception of the absence of nitrogen. In contrast, previous studies have shown that temperature and light stress induced the akinete formation by day 11 (Moore et al., 2005; Yamamoto and Shiah, 2014). In *Anabaena cylindrica*, visible akinete appeared around day 11 under nitrogen starvation (Hori et al., 2002) and day seven under P limitation conditions (Olli et al., 2005). Notably, *Anabaena iyengarii* and *Westiellopsis prolifica* required up to 45 days to akinete formation (Agrawal and Misra, 2002). Our findings show that nitrogen limitation is a very efficient inducer for akinete formation in *R. raciborskii,* which might contribute to its global invasion success.

### 4.2 Temperature effects on akinete formation

Temperature effects were not consistent among strains; while some strains (mainly 26D9 and 27F11) had lower akinete formation at 28 °C than at 20 °C, and MEL07 and ZIE11 showed no or the opposite response(Figure 2). These findings are consistent with field observation. For example, *R. raciborskii* showed increased akinete formation at 20 °C (Mehnert et al., 2014), and similar results were observed in Lake Kinneret, where akinete formation increased at temperatures below 25 °C (Alster et al., 2010). In natural *R. raciborskii* populations, akinete formation typically occurs during warm summer months when the species becomes dominant (Padisák, 2003; Padisák and Istvánovics, 1997; Yamamoto and Shiah, 2014). Some tropical strains from Brazil ponds do not form akinetes below 21 °C (Bittencourt-Oliveira et al., 2012). While previous studies on temperate strains suggest the optimal akinete formation occurs between 24 and 30 °C, our results indicate that the response is strain-dependent. Some tropical species persist throughout the year and do not form akinetes in the field (Everson et al., 2011). At the same time, vegetative survival at low temperatures has been reported for some temperate strains. For example, Dokulil (2016) found that *R. raciborskii* can survive at low temperature in Austrian lakes, though growth ceases below ~11 °C. This suggest that while certain populations can overwinter vegetatively, many rely on akinete formation during warmer periods to withstand prolonged exposure to suboptimal winter temperatures. Together, these findings support the view that temperature-dependent akinete formation is highly strain-specific, reflecting the ecological and evolutionary histories of individual populations.

### 4.3 Strain-specific resource strategies

Strains differed in the scale and timing of akinete formation, reflecting diverse survival strategies that range from rapid response to delayed investment. Since we cannot exclude that some filaments within a population contributed to vegetative growth and others for akinete formation, we discuss our findings on a population level. For instance, strain MEL07 reached an early peak in akinete formation by day 9, suggesting a fast-response strategy, whereas strain 27F11 exhibited a delayed but more extensive investment in dormant stages. Additionally, contrasting patterns of akinete abundance per milligram of carbon among strains suggest distinct strategies for resource allocation. Generally, akinete formation involves significant metabolic adjustments. Large amounts of carbon and nitrogen are invested in the akinete envelope, while carbon fixation may be reduced and cell division is temporarily interrupted (Sukenik et al., 2018), in most cases, growth tends to decline during akinete formation (Cirés et al., 2013). In *Nostoc punctiforme*, for instance, energy availability becomes limited during akinete differentiation, highlighting the energetic cost of this process (Meeks et al., 2002). Temperature modulated the coupling between growth and differentiation, but patterns remained strain-dependent. Strains 27F11 and ZIE11 maintained active growth while forming akinetes, indicating a simultaneous allocation strategy that support both proliferation and persistence. Conversely, strains MEL07 and 26D9 showed a more sequential pattern, investing in akinete formation after biomass accumulation, representing a delayed-investment strategy. Such contrasting strategies reflect high physiological flexibility, which enhances ecological resilience and invasion potential of *R. raciborskii* under fluctuating conditions. Overall, *R. raciborskii* strains were able to use both strategies under nitrogen-limiting conditions, suggesting effective nitrogen fixation, which may support early akinete development (Ahuja et al., 2008). The four strains in the present study form heterocysts also at nitrogen replete conditions (Bolius et al., 2017), further indicating a high capacity for physiological plasticity.

The C: N ratio among *R. raciborskii* strains remained constant close to 5, indicating proportionate carbon and nitrogen uptake throughout the experiments. Experiments in *Anabaena torulosa* demonstrated that a C: N ratio of 5 resulted in higher abundance of akinete, whereas, akinete formation was inhibited at a C: N ratio of 7 (Ahuja et al., 2008). In our study, phosphorus was not a limiting factor, but it is important to note that the response to phosphorus availability can vary among cyanobacterial species. *R. raciborskii* specifically requires phosphorus-replete conditions for both growth and akinete formation (Moore et al., 2003, 2005). When phosphorus is limited, akinete formation in *R. raciborskii* is inhibited (Sprőber et al., 2003). Altogether, these findings emphasize the importance of resource-dependent and strain-specific strategies.

Although akinete formation is often discussed in the context of invasion ecology, our experiment did not quantify invasion success directly. Nonetheless, previous mesocosm experiments with the same four strains allow for a cautions comparison. Linking akinete formation with invasion success measured through vegetative growth could suggest a potential trade-off, where strong invaders invest less in dormant stages and weaker invaders invest more. However, the comparable akinete formation in the most (ZIE11) and least (26D9) successful strains (Bolius et al., 2020) does not support such a trade-off under the tested conditions. This reinforces the conclusion that multiple, context-dependent traits beyond akinete formation likely shape invasion success in *R. raciborskii*.

### 4.4 Variation in akinete maturation

The low abundance of mature akinetes observed in our study suggests a delay between the initiation and full development of the akinetes. In MEL07 and ZIE11, most akinetes remained immature at both temperatures, whereas strain 27F11 exhibited a higher proportion of mature akinetes, although never exceeding 55%. In contrast, strain 26D9 showed a high proportion of mature akinetes at 20 °C, which was lower at 28 °C (Figure 2). These differences suggest that both timing and completion of akinete formation vary among strains even under identical conditions. We can only speculate about the reason behind the relatively high number of immature akinetes. Maybe the metabolism under severe nitrogen limitation is truncated and some essential biochemical compounds are lacking. In *Anabaena*, prolonged phosphate starvation resulted in delayed maturation, with only ~70% of akinetes mature after 30 days, showing that severe nutrient limitation might increase the time required for full maturation (Sutherland et al., 1979). In our study, mature akinetes seldom exceeded 25% in MEL07 and ZIE11, suggesting that longer periods may be required for full maturation. The marked differences among strains underscore high intraspecific variability of *R. raciborskki* and highlight the necessity of study multiple strains to draw general conclusions. This diversity may also facilitate invasion: when multiple strains colonize a new habitat, the probability that at least one possesses traits conducive to establishment increases (Bolius et al., 2020).

### 4.5 Akinete size differentiation

The akinetes were typically found in the proximity of heterocysts which is consistent with previous reports (Alster et al., 2010; Moore et al., 2003; Stevic et al., 2025), however, among the four strains, akinetes differed in length. Most previous studies measured akinetes within the filament, i.e., during the maturation process (Bittencourt-Oliveira et al., 2012; Komárková et al., 1999; Stevic et al., 2025). In contrast, our measurements were taken from fully mature akinetes, which were already detached and ready to enter dormancy. Therefore, our data are not directly comparable to those earlier studies and this explains at least partly the long akinetes of our strains. In a study of *R. raciborskii* in Lake Kinneret, Israel, mature akinetes ranged from 7 to 18 μm in length, and 3 to 6 μm in width (Alster et al., 2010). In our study akinetes were substantially larger, ranging from 19.6 to 34.7 μm in length showing a substantial resource allocation. One might expect a trade-off between size and abundance, but no consistent trend was found between the abundance of mature akinetes and their size. Two strains showed contrasting responses— strain 27F11 showed high akinete abundance at 20 °C despite their smaller size, whereas strain 26D9 formed fewer but larger akinetes at 28 °C. A general trend toward smaller akinetes at higher temperature (28 °C) was observed, suggesting a temperature-dependence on akinete size. This is consistent with previous findings in *Nodularia spumigena*, where elevated temperatures (30 °C) also led to reduce akinetes size (Silveira and Odebrecht, 2019). Akinetes can vary in size and differentiation stages within the same trichome, indicating an unsynchronized akinete formation (Maldener et al., 2014).

The ecological significance of akinete size and maturity remains poorly understood. Previous studies have not established clear links between akinete size and persistence or germination likelihood, and our results did not reveal such a relationship either. However, because akinetes store nitrogen and other reserves mobilized during germination, whereas vegetative growth depends on freshly fixed nutrients (Garg and Maldener, 2021; Perez et al., 2018), their physiological state and degree of maturity likely influence survival under adverse conditions and may ultimately affect colonization success.

### 4.6 Akinete formation and its ecological role

The high intraspecific variation among *R. raciborskii* strains likely supports their adaptability and successful establishment in new environments. During the invasion process, Cyanobacteria— particularly diazotrophic species—can disperse and proliferate in the water column through filament fragmentation, surviving a few weeks or months when temperature and light levels decline during the autumn season (Adams and Duggan, 1999). Akinete formation is therefore crucial for long-term persistence. Several case studies illustrate the ecological importance of akinetes. In Lake Balaton (Hungary), *R. raciborskii* initially appeared in low abundance, but establishment by producing more akinetes than native Nostocales (Padisák and Istvánovics, 1997). In German lakes, although vegetative abundance of *R. raciborskii* is often lower than *Aphanizomenon gracile*; *R. raciborskii* compensates for this by producing a comparable or even greater number of akinetes (Mehnert et al., 2014). In Lake Melangsee, continuous persistence of *R. raciborskii* is attributed to sediment inoculum formed by akinetes (“seed bank”), which facilitates its establishment in the ecosystem (Rücker et al., 2009). In Lake Nero (Russia), the authors presume that *R. raciborskii* was likely introduced during a warm period. Although blooms did not occur for several years due to persistently low lake temperatures, the strain was able to form akinete and re-establish high abundance when conditions became favorable again (Sidelev et al., 2020). A similar phenomenon has been observed in the Baltic Sea, where akinete formation in *Anabaena* influences bloom dynamics (Olli et al., 2005).

Our results support the existence of contrasting strategies: rapid-response strains that form akinetes quickly but in lower abundance, and delayed-investment strains that form many akinetes more slowly. Temperature modulated both abundance and timing. At 20 °C, akinete formation occurred more rapidly but in lower abundance, suggestive of a survival strategy under suboptimal conditions. Being of tropical origin, optimal growth of *R. raciborskii* occurs at higher temperature, enabling vegetative persistence before switching to akinete formation. These strategies promote resilience and enhance invasion success under fluctuating conditions. Environmental variability, particularly nutrient levels and temperatures, strongly affects bloom timing and intensity (Cottingham et al., 2021). Under prolonged nutrient limitation, akinetes may remain dormant and suppress bloom recurrence (Karlsson-Elfgren and Brunberg, 2004; Moore et al., 2003). Increasing phosphorus enrichment and rising temperatures, however, may favor both germination and vegetative growth(Carey et al., 2012; Cottingham et al., 2021). Maintaining low nutrient concentration, particularly phosphorous, would therefore limit the reactivation of akinetes and help prevent bloom resurgence. Because many strains tolerate high temperatures, climate warming may counteract nutrient-reduction strategies and promote future blooms (Sinha et al., 2012; Wiedner et al., 2007). As global temperature continue to rise and fluctuate, *R. raciborskii* is likely gain a competitive advantage, further expanding into new ecosystems. Its ability to thrive under nutrient-limited conditions and outcompete other cyanobacteria and phytoplankton groups has been well documented (Zheng et al., 2023). These findings align with previous studies showing that the invasion potential of *R. raciborskii* is strain dependent (Bolius et al., 2017, 2020). However, in those experiments, only the planktonic phase was studied. The findings of the present study extend the concept of intraspecific variability in planktonic-phase traits (Bolius et al., 2020; Weithoff and Stefan, 2024; Willis, 2025) to the benthic phase, highlighting resting stages as a potential mechanism for successful invasion.

Ultimately, in a natural environment, akinete formation appears to be regulated by a complex interplay of several factors including temperature and nutrient ratios. However, since we found substantial strain-specific differences, it can be expected that under different global change scenarios, the likelihood of one or a few successful strains of *R. raciborskii* in a given area is quite high preserving populations in invaded regions.

## Data Availability

The original contributions presented in the study are included in the article/Supplementary material, further inquiries can be directed to the corresponding author.

## References

[ref1] Adams D. G. Duggan P. S. (1999). Heterocyst and akinete differentiation in cyanobacteria. New Phytol. 144, 3–33. doi: 10.1046/j.1469-8137.1999.00505.x

[ref2] Agrawal S. C. Misra U. (2002). Vegetative survival, akinete and zoosporangium formation and germination in some selected algae as affected by nutrients, pH, metals, and pesticides. Folia Microbiol. 47, 527–534. doi: 10.1007/BF02818793, 12503399

[ref3] Aguilera A. Gómez E. B. Kaštovský J. Echenique R. O. Salerno G. L. (2018). The polyphasic analysis of two native *Raphidiopsis* isolates supports the unification of the genera *Raphidiopsis* and *Cylindrospermopsis* (Nostocales, Cyanobacteria). Phycologia 57, 130–146. doi: 10.2216/17-2.1

[ref4] Ahuja G. Khattar J. S. Sarma T. A. (2008). Interaction between carbon and nitrogen metabolism during akinete development in the cyanobacterium *Anabaena torulosa*. J. Basic Microbiol. 48, 125–129. doi: 10.1002/jobm.200700302, 18383224

[ref5] Alster A. Kaplan-Levy R. N. Sukenik A. Zohary T. (2010). Morphology and phylogeny of a non-toxic invasive *Cylindrospermopsis raciborskii* from a Mediterranean lake. Hydrobiologia 639, 115–128. doi: 10.1007/s10750-009-0044-y

[ref6] Alvarez Dalinger F. S. Borja C. N. Muñoz C. Moraña L. B. Lozano V. L. (2024). Invasive freshwater algae and cyanobacteria are overlooked: insights from a bibliometric study. Hydrobiologia 852, 2277–2292. doi: 10.1007/s10750-024-05655-7

[ref7] Bittencourt-Oliveira M. Buch B. Hereman T. Arruda-Neto J. Moura A. Zocchi S. (2012). Effects of light intensity and temperature on *Cylindrospermopsis raciborskii* (Cyanobacteria) with straight and coiled trichomes: growth rate and morphology. Braz. J. Biol. 72, 343–351. doi: 10.1590/s1519-69842012000200016, 22735143

[ref8] Bolius S. Morling K. Wiedner C. Weithoff G. (2020). Genetic identity and herbivory drive the invasion of a common aquatic microbial invader. Front. Microbiol. 11:1598. doi: 10.3389/fmicb.2020.01598, 32754141 PMC7370804

[ref9] Bolius S. Wiedner C. Weithoff G. (2017). High local trait variability in a globally invasive cyanobacterium. Freshw. Biol. 62, 1879–1890. doi: 10.1111/fwb.13028

[ref10] Bonilla S. Aubriot L. Soares M. C. S. González-Piana M. Fabre A. Huszar V. L. M. . (2012). What drives the distribution of the bloom-forming cyanobacteria *Planktothrix agardhii* and *Cylindrospermopsis raciborskii*? FEMS Microbiol. Ecol. 79, 594–607. doi: 10.1111/j.1574-6941.2011.01242.x, 22092489

[ref11] Carey C. C. Ibelings B. W. Hoffmann E. P. Hamilton D. P. Brookes J. D. (2012). Eco-physiological adaptations that favour freshwater cyanobacteria in a changing climate. Water Res. 46, 1394–1407. doi: 10.1016/j.watres.2011.12.016, 22217430

[ref12] Chonudomkul D. Yongmanitchai W. Theeragool G. Kawachi M. Kasai F. Kaya K. . (2004). Morphology, genetic diversity, temperature tolerance and toxicity of *Cylindrospermopsis raciborskii* (Nostocales, Cyanobacteria) strains from Thailand and Japan. FEMS Microbiol. Ecol. 48, 345–355. doi: 10.1016/j.femsec.2004.02.014, 19712304

[ref13] Cirés S. Wörmer L. Wiedner C. Quesada A. (2013). Temperature-dependent dispersal strategies of *Aphanizomenon ovalisporum* (Nostocales, Cyanobacteria): implications for the annual life cycle. Microb. Ecol. 65, 12–21. doi: 10.1007/s00248-012-0109-8, 22915156

[ref14] Cottingham K. L. Weathers K. C. Ewing H. A. Greer M. L. Carey C. C. (2021). Predicting the effects of climate change on freshwater cyanobacterial blooms requires consideration of the complete cyanobacterial life cycle. J. Plankton Res. 43, 10–19. doi: 10.1093/plankt/fbaa059

[ref15] Dokulil M. T. (2016). Vegetative survival of *Cylindrospermopsis raciborskii* (Cyanobacteria) at low temperature and low light. Hydrobiologia 764, 241–247. doi: 10.1007/s10750-015-2228-y

[ref16] Dolman A. M. Mischke U. Wiedner C. (2016). Lake-type-specific seasonal patterns of nutrient limitation in German lakes, with target nitrogen and phosphorus concentrations for good ecological status. Freshw. Biol. 61, 444–456. doi: 10.1111/fwb.12718

[ref17] Everson S. Fabbro L. Kinnear S. Wright P. (2011). Extreme differences in akinete, heterocyte and cylindrospermopsin concentrations with depth in a successive bloom involving *Aphanizomenon ovalisporum* (Forti) and *Cylindrospermopsis raciborskii* (Woloszynska) Seenaya and Subba Raju. Harmful Algae 10, 265–276. doi: 10.1016/j.hal.2010.10.006

[ref18] Fay P. Lynn J. A. Majer S. C. (1984). Akinete development in the planktonic blue-green alga *Anabaena circinalis*. Br. Phycol. J. 19, 163–173. doi: 10.1080/00071618400650171

[ref19] Fuentes N. Ríos-Henríquez C. Díaz P. A. (2022). Hydroclimatic drivers associated with an unusual bloom of *Microcystis aeruginosa* and increase of CyanoHABs in a deep oligotrophic lake. J. Plankton Res. 44, 68–72. doi: 10.1093/plankt/fbab079

[ref20] Garg R. Maldener I. (2021). The formation of spore-like akinetes: a survival strategy of filamentous cyanobacteria. Microb. Physiol. 31, 296–305. doi: 10.1159/000517443, 34482304

[ref21] Guillard R. R. L. Lorenzen C. J. (1972). Yellow-green algae with chlorophyllide c. J. Phycol. 8, 10–14.

[ref22] Hamilton P. B. Ley L. M. Dean S. Pick F. R. (2005). The occurrence of the cyanobacterium *Cylindrospermopsis raciborskii* in Constance Lake: an exotic cyanoprokaryote new to Canada. Phycologia 44, 17–25. doi: 10.2216/0031-8884(2005)44[17:TOOTCC]2.0.CO;2

[ref23] Haubrock P. J. Soto I. Cano-Barbacil C. Theissinger K. Rios-Henriquez C. Parker B. . (2025). Germany’s established non-native species: a comprehensive breakdown. Environ. Sci. Eur. 37:56. doi: 10.1186/s12302-025-01094-w

[ref24] Ho H. I. Park C. H. Yoo K. E. Kim N. Y. Hwang S. J. (2024). Survival and development strategies of Cyanobacteria through akinete formation and germination in the life cycle. Water 16, 1–22. doi: 10.3390/w16050770

[ref25] Holdich D. M. Reynolds J. D. Souty-Grosset C. Sibley P. J. (2009). A review of the ever increasing threat to European crayfish from non-indigenous crayfish species. Knowl. Manag. Aquat. Ecosyst. 11, 394–395. doi: 10.1051/kmae/2009025

[ref26] Hori K. Ishii S. Ikeda G. Okamoto J. Tanji Y. Weeraphasphong C. . (2002). Behavior of filamentous cyanobacterium *Anabaena* spp. in water column and its cellular characteristics. Biochem. Eng. J. 10, 217–225. doi: 10.1016/S1369-703X(01)00185-1

[ref27] Kaplan-Levy R. N. Hadas O. Summers M. L. Sukenik A. (2010). “Akinetes: dormant cells of cyanobacteria” in Dormancy and resistance in harsh environments. eds. Lubzens E. Cerda J. Clark M., Berlin, Heidelberg: Springer-Verlag, 21, 5–27.

[ref28] Karlsson-Elfgren I. Brunberg A. K. (2004). The importance of shallow sediments in the recruitment of *Anabaena* and *Aphanizomenon* (Cyanophyceae). J. Phycol. 40, 831–836. doi: 10.1111/j.1529-8817.2004.04070.x

[ref29] Komárková J. Laudares-Silva R. Senna P. A. C. (1999). Extreme morphology of *Cylindrospermopsis raciborskii* (Nostocales, Cyanobacteria) in the Lagoa do peri, a freshwater coastal lagoon, Santa Catarina, Brazil. Algol. Stud. 94, 207–222. doi: 10.1127/algol_stud/94/1999/207

[ref30] Legrand B. Miras Y. Beauger A. Dussauze M. Latour D. (2019). Akinetes and ancient DNA reveal toxic cyanobacterial recurrences and their potential for resurrection in a 6700-year-old core from a eutrophic lake. Sci. Total Environ. 687, 1369–1380. doi: 10.1016/j.scitotenv.2019.07.100, 31412470

[ref31] Litchman E. (2010). Invisible invaders: non-pathogenic invasive microbes in aquatic and terrestrial ecosystems. Ecol. Lett. 13, 1560–1572. doi: 10.1111/j.1461-0248.2010.01544.x, 21054733

[ref32] Maldener I. Summers M. L. Sukenik A. (2014). “Cellular differentiation in filamentous cyanobacteria” in The cell biology of Cyanobacteria. eds. Flores E. Herrero A. (Poole: Caister Academic Press), 263–291.

[ref33] Meeks J. C. Campbell E. L. Summers M. L. Wong F. C. (2002). Cellular differentiation in the cyanobacterium *Nostoc punctiforme*. Arch. Microbiol. 178, 395–403. doi: 10.1007/s00203-002-0476-5, 12420158

[ref34] Mehnert G. Rucker J. Wiedner C. (2014). Population dynamics and akinete formation of an invasive and a native cyanobacterium in temperate lakes. J. Plankton Res. 36, 378–387. doi: 10.1093/plankt/fbt122

[ref35] Moore D. O’Donohue M. Garnett C. Critchley C. Shaw G. (2005). Factors affecting akinete differentiation in *Cylindrospermopsis raciborskii* (Nostocales, Cyanobacteria). Freshw. Biol. 50, 345–352. doi: 10.1111/j.1365-2427.2004.01324.x

[ref36] Moore D. O’Donohue M. Shaw G. Critchley C. (2003). Potential triggers for akinete differentiation in an Australian strain of the cyanobacterium *Cylindrospermopsis raciborskii* (AWT 205/1). Hydrobiologia 506–509, 175–180. doi: 10.1023/B:HYDR.0000008536.01716.1a

[ref37] Olli K. Kangro K. Kabel M. (2005). Akinete production of *Anabaena lemmermannii* and *A. cylindrica* (Cyanophyceae) in natural populations of N- and P-limited coastal mesocosms. J. Phycol. 41, 1094–1098. doi: 10.1111/j.1529-8817.2005.00153.x

[ref38] Padisák J. (1997). *Cylindrospermopsis raciborskii* (Woloszyska) Seenayya et Subba Raju, an expanding, highly adaptive cyanobacterium: worldwide distribution and review of its ecology. Arch. Hydrobiol. Suppl. Monogr. Beitr. 107, 563–593.

[ref39] Padisák J. (2003). Estimation of minimum sedimentary inoculum (akinete) pool of *Cylindrospermopsis raciborskii*: a morphology and life-cycle based method. Hydrobiologia 502, 389–394. doi: 10.1023/B:HYDR.0000004296.49074.0a

[ref40] Padisák J. Istvánovics V. (1997). Differential response of blue-green algal groups to phosphorus load reduction in a large shallow lake: Balaton, Hungary. SIL Proc. 26, 574–580. doi: 10.1080/03680770.1995.11900783

[ref41] Padisák J. Vasas G. Borics G. (2016). Phycogeography of freshwater phytoplankton: traditional knowledge and new molecular tools. Hydrobiologia 764, 3–27. doi: 10.1007/s10750-015-2259-4

[ref42] Perez R. Forchhammer K. Salerno G. Maldener I. (2016). Clear differences in metabolic and morphological adaptations of akinetes of two nostocales living in different habitats. Microbiology 162, 214–223. doi: 10.1099/mic.0.000230, 26679176

[ref43] Perez R. Wörmer L. Sass P. Maldener I. (2018). A highly asynchronous developmental program triggered during germination of dormant akinetes of filamentous diazotrophic cyanobacteria. FEMS Microbiol. Ecol. 94, 1–11. doi: 10.1093/FEMSEC/FIX131, 29228342

[ref44] Pyšek P. Hulme P. E. Simberloff D. Bacher S. Blackburn T. M. Carlton J. T. . (2020). Scientists’ warning on invasive alien species. Biol. Rev. 95, 1511–1534. doi: 10.1111/brv.12627, 32588508 PMC7687187

[ref45] Ramm J. Rücker J. Knie M. Nixdorf B. (2017). Lost in the dark: estimation of the akinete pool for the recruitment of Nostocales populations (cyanobacteria) in a temperate deep lake. J. Plankton Res. 39, 392–403. doi: 10.1093/plankt/fbx010

[ref46] Reinl K. L. Harris T. D. North R. L. Almela P. Berger S. A. Bizic M. . (2023). Blooms also like it cold. Limnol. Oceanogr. Lett. 8, 546–564. doi: 10.1002/lol2.10316

[ref47] Rücker J. Tingwey E. I. Wiedner C. Anu C. M. Nixdorf B. (2009). Impact of the inoculum size on the population of Nostocales cyanobacteria in a temperate lake. J. Plankton Res. 31, 1151–1159. doi: 10.1093/plankt/fbp067

[ref48] Sala O. E. Chapin F. S. Armesto J. J. Berlow E. Bloomfield J. Dirzo R. . (2000). Global biodiversity scenarios for the year 2100. Science 287, 1770–1774. doi: 10.1126/science.287.5459.1770, 10710299

[ref9001] Seenayya G. Subba Raju N. S. (1972). On the ecology and syste-matic position of the alga known as Anabaenopsis raciborskii (Wolosz.) Elenk. and a critical evaluation of the forms described under the genus Anabaenopsis. In: Taxonomy and biology of blue-green algae, ed. T.V. Desikachary. Centre for Advanced Study in Botany, Univ. Madras. Madras, 52–57.

[ref49] Sidelev S. Koksharova O. Babanazarova O. Fastner J. Chernova E. Gusev E. (2020). Phylogeographic, toxicological and ecological evidence for the global distribution of *Raphidiopsis raciborskii* and its northernmost presence in Lake Nero, Central Western Russia. Harmful Algae 98:101889. doi: 10.1016/j.hal.2020.101889, 33129449

[ref50] Silveira S. B. Odebrecht C. (2019). Effects of salinity and temperature on the growth, toxin production, and akinete germination of the cyanobacterium *Nodularia spumigena*. Front. Mar. Sci. 6, 1–12. doi: 10.3389/fmars.2019.0033936817748

[ref51] Singh P. Khan A. Srivastava A. (2020). “Heterocyst and akinete differentiation in cyanobacteria: a view toward cyanobacterial symbiosis” in Advances in cyanobacterial biology. ed. Whitton B. A. (Cambridge: Academic Press), 235–248.

[ref52] Sinha R. Pearson L. A. Davis T. W. Burford M. A. Orr P. T. Neilan B. A. (2012). Increased incidence of *Cylindrospermopsis raciborskii* in temperate zones - is climate change responsible? Water Res. 46, 1408–1419. doi: 10.1016/j.watres.2011.12.019, 22284981

[ref53] Smith V. H. (1983). Low nitrogen to phosphorus ratios favor dominance by blue-green algae in lake phytoplankton. Science 221, 669–671. doi: 10.1126/science.221.4611.669, 17787737

[ref54] Sprőber P. Shafik H. M. Présing M. Kovács A. W. Herodek S. (2003). Nitrogen uptake and fixation in the cyanobacterium *Cylindrospermopsis raciborskii* under different nitrogen conditions. Hydrobiologia 506-509, 169–174. doi: 10.1023/B:HYDR.0000008617.90245.5f, 11099962

[ref55] Stevic F. Mihaljevic M. Maronic D. Š. Pfeiffer T. Ž. Zahirovic V. (2025). Morphological variability of a natural population of cyanobacterium *Raphidiopsis raciborskii* in a temperate floodplain lake. Taxon 5:16. doi: 10.3390/taxonomy5020016

[ref56] Strayer D. L. (2009). Twenty years of zebra mussels: lessons from the mollusk that made headlines. Front. Ecol. Environ. 7, 135–141. doi: 10.1890/080020

[ref57] Sukenik A. Hadas O. Kaplan A. Quesada A. (2012). Invasion of Nostocales (cyanobacteria) to subtropical and temperate freshwater lakes - physiological, regional, and global driving forces. Front. Microbiol. 3, 1–9. doi: 10.3389/fmicb.2012.00086, 22408640 PMC3297820

[ref58] Sukenik A. Quesada A. Salmaso N. (2015). Global expansion of toxic and non-toxic cyanobacteria: effect on ecosystem functioning. Biodivers. Conserv. 24, 889–908. doi: 10.1007/s10531-015-0905-9

[ref59] Sukenik A. Rücker J. Maldener I. (2018). “Dormant cells (akinetes) of filamentous Cyanobacteria demonstrate a great variability in morphology, physiology, and ecological function” in Cyanobacteria: from basic science to applications. eds. Mishra A. K. Tiwari D. N. Rai A. N. (Amsterdam: Elsevier), 65–91.

[ref60] Sutherland J. M. Herdman M. Stewart W. D. P. (1979). Akinetes of the cyanobacterium *Nostoc* PCC 7524: macromolecular composition, structure and control of differentiation. J. Gen. Microbiol. 115, 273–287. doi: 10.1099/00221287-115-2-273

[ref61] Turpin D. H. Edie S. A. Canvin D. T. (1984). In vivo nitrogenase regulation by ammonium and methylamine and the effect of MSX on ammonium transport in *Anabaena flos-aquae*. Plant Physiol. 74, 701–704. doi: 10.1104/pp.74.3.701, 16663484 PMC1066749

[ref62] Weithoff G. Stefan M. B. (2024). Weak effect of temperature fluctuations on the invasion of *Raphidiopsis raciborskii* (Cyanobacteria) in experimental plankton microcosms. J. Phycol. 61, 261–266. doi: 10.1111/jpy.13536, 39652370 PMC12044404

[ref63] Wejnerowski Ł. Sikora-Koperska A. Dawidowicz P. (2020). Temperature elevation reduces the sensitivity of invasive cladoceran *Daphnia lumholtzi* to filamentous cyanobacterium *Raphidiopsis raciborskii*. Freshw. Biol. 65, 935–946. doi: 10.1111/fwb.13480

[ref64] Welschmeyer N. A. (1994). Fluorometric analysis of chlorophyll a in the presence of chlorophyll b and pheopigments. Limnol. Oceanogr. 39, 1985–1992. doi: 10.4319/lo.1994.39.8.1985

[ref65] Wiedner C. Rücker J. Brüggemann R. Nixdorf B. (2007). Climate change affects timing and size of populations of an invasive cyanobacterium in temperate regions. Oecologia 152, 473–484. doi: 10.1007/s00442-007-0683-5, 17375336

[ref66] Wilkinson G. M. Walter J. A. Buelo C. D. Pace M. L. (2022). No evidence of widespread algal bloom intensification in hundreds of lakes. Front. Ecol. Environ. 20, 16–21. doi: 10.1002/fee.2421

[ref67] Willis A. (2025). Intraspecific diversity in *Raphidiopsis raciborskii*: a key to its invasive success. J. Phycol. 61, 258–260. doi: 10.1111/jpy.70011, 40308159

[ref68] Willis A. Chuang A. W. Burford M. A. (2016). Nitrogen fixation by the diazotroph *Cylindrospermopsis raciborskii* (Cyanophyceae). J. Phycol. 52, 854–862. doi: 10.1111/jpy.12451, 27440068

[ref69] Wood S. A. Pochon X. Luttringer-Plu L. Vant B. N. Hamilton D. P. (2014). Recent invader or indicator of environmental change? A phylogenetic and ecological study of *Cylindrospermopsis raciborskii* in New Zealand. Harmful Algae 39, 64–74. doi: 10.1016/j.hal.2014.06.013

[ref70] Woolway R. I. Sharma S. Weyhenmeyer G. A. Debolskiy A. Golub M. Mercado-Bettín D. . (2021). Phenological shifts in lake stratification under climate change. Nat. Commun. 12, 1–11. doi: 10.1038/s41467-021-22657-4, 33875656 PMC8055693

[ref71] Yamamoto Y. Shiah F. K. (2014). Growth, trichome size and akinete production of *Cylindrospermopsis raciborskii* (cyanobacteria) under different temperatures: comparison of two strains isolated from the same pond. Phycol. Res. 62, 147–152. doi: 10.1111/pre.12040

[ref72] Zhang C. C. Laurent S. Sakr S. Peng L. Bédu S. (2006). Heterocyst differentiation and pattern formation in cyanobacteria: a chorus of signals. Mol. Microbiol. 59, 367–375. doi: 10.1111/j.1365-2958.2005.04979.x, 16390435

[ref73] Zheng L. Liu Y. Li R. Yang Y. Jiang Y. (2023). Recent advances in the ecology of bloom-forming *Raphidiopsis* (*Cylindrospermopsis*) *raciborskii*: expansion in China, intraspecific heterogeneity and critical factors for invasion. Int. J. Environ. Res. Public Health 20:1984. doi: 10.3390/ijerph20031984, 36767351 PMC9915880

