# Supplementary material for: Akinete formation under nitrogen limitation in an invasive cyanobacterium
Source: Front Microbiol. 2025 Dec 12;16:1677844. doi: 10.3389/fmicb.2025.1677844 (PMC12742475; doi:10.3389/fmicb.2025.1677844)
Supplement: Supplementary file 1 [file Data_Sheet_1.docx]

Supplementary Material

## Supplementary Figures


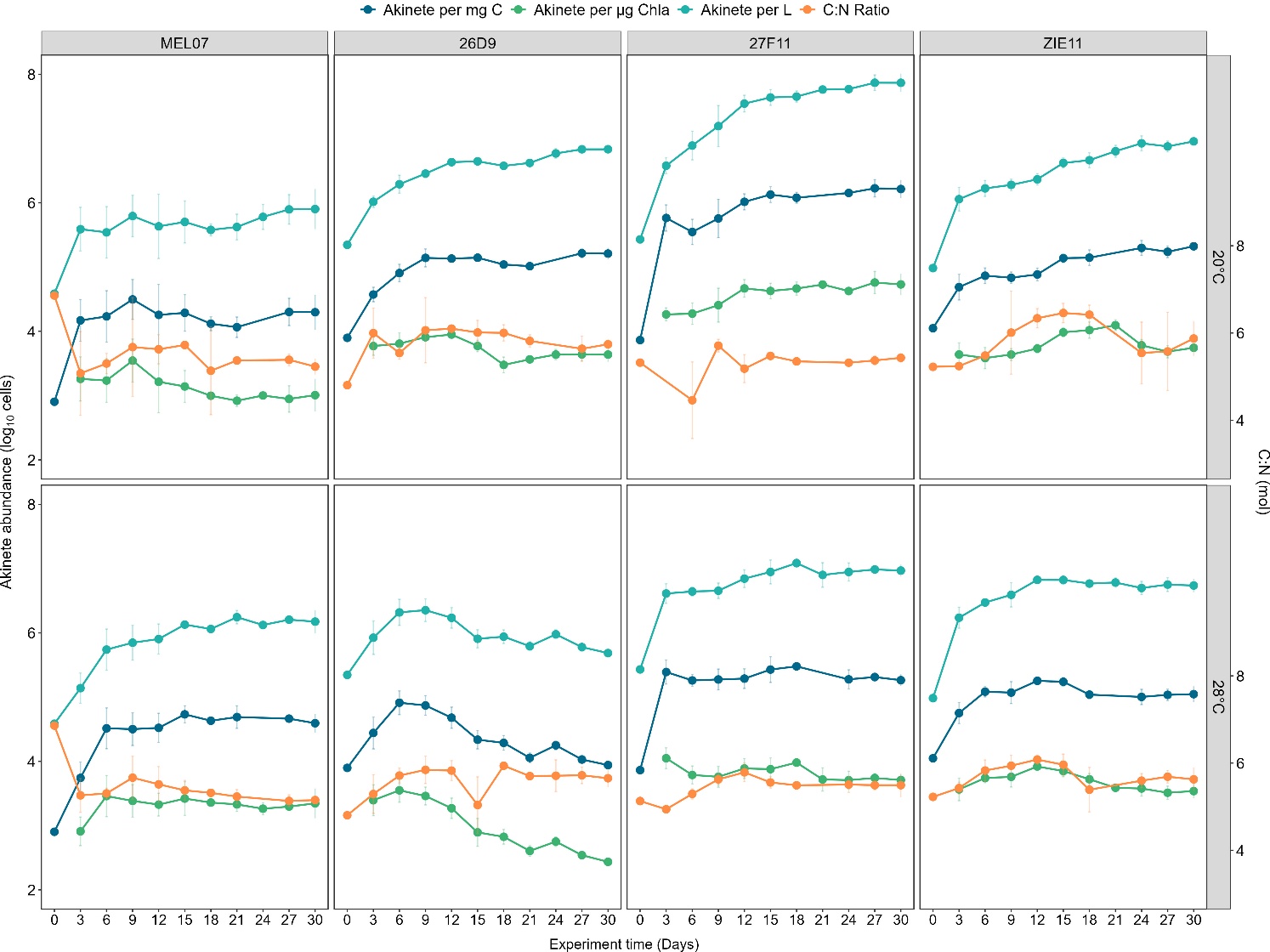


Supplementary Figure 1: Temporal dynamics of akinete abundance and C:N ratio in four *R. raciborskii* strains (MEL07, 26D9, 27F11, and ZIE11) under temperatures (20°C and 28°C) and nitrogen free conditions. Akinete abundance is expressed as a akinete per mg C (blue), akinetes per µg Chl-*a* (green), and the molar C:N ratio (orange). Symbols and error bars indicate mean values ± SD. Panels are arranged by strain (columns) and treatments (rows).


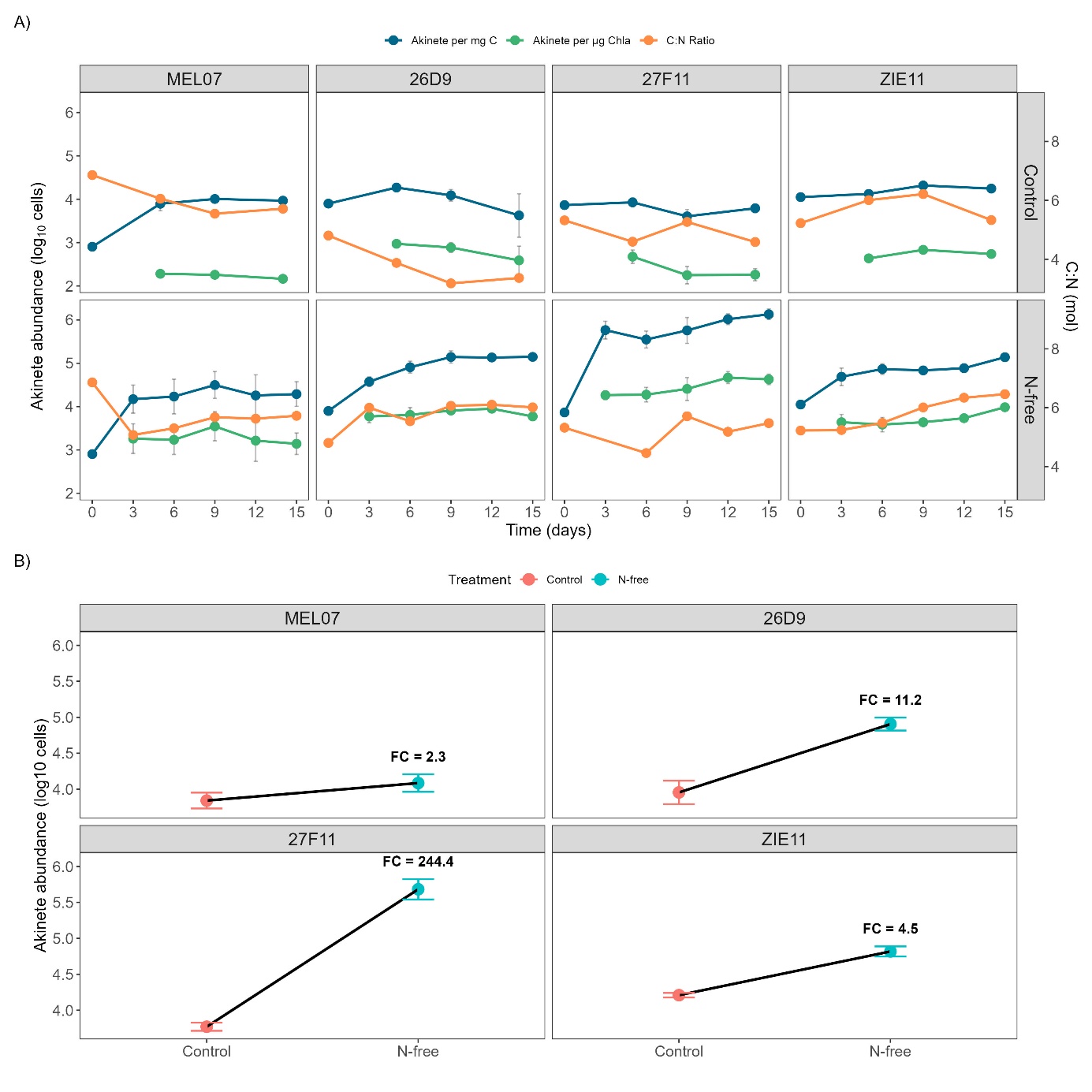


Supplementary Figure 2: A) Temporal dynamics of akinete abundance and C:N ratio in four *R. raciborskii* strains (MEL07, 26D9, 27F11, and ZIE11) under control and nitrogen-free conditions (N-free). Akinete abundance is expressed as akinete per mg C (blue), akinetes per µg Chl-*a* (green), and the molar C:N ratio (orange). Panels are arranged by strain (columns) and treatments (rows).

B) Akinete abundance per mg C was used to determine differences between the control and N-free treatments, with fold-change indicated for each strain. Control data were obtained at 20°C from an independent experiment; therefore, comparisons with N-free cultures are limited to 20°C, as no control measurements were available at 28°C. Only the first 15 days are showed. Symbols and error bars indicate mean values ± SD.
